# Single Particle Assays to Determine Heterogeneities within Fluid Catalytic Cracking Catalysts

**DOI:** 10.1002/chem.201905880

**Published:** 2020-05-29

**Authors:** Anne‐Eva Nieuwelink, Marjolein E. Z. Velthoen, Yoni C. M. Nederstigt, Kristel L. Jagtenberg, Florian Meirer, Bert M. Weckhuysen

**Affiliations:** ^1^ Inorganic Chemistry and Catalysis Debye Institute for Nanomaterials Science Utrecht University Universiteitsweg 99 3584 CG Utrecht The Netherlands

**Keywords:** deactivation, density separation, fluid catalytic cracking, heterogeneity, spectroscopy

## Abstract

Fluid catalytic cracking (FCC) is an important process in oil refinery industry to produce gasoline and propylene. Due to harsh reaction conditions, FCC catalysts are subject to deactivation through for example, metal accumulation and zeolite framework collapse. Here, we perform a screening of the influence of metal poisons on the acidity and accessibility of an industrial FCC catalyst material using laboratory‐based single particle characterization that is, μ‐XRF and fluorescence microscopy in combination with probe molecules. These methods have been performed on density‐separated FCC catalyst fractions, allowing to determine interparticle heterogeneities in the catalyst under study. It was found that with increasing catalyst density and metal content, the acidity and accessibility of the catalyst particles decreased, while their distribution narrowed with catalyst age. For example, particles containing high Ni level possessed very low acidity and were hardly accessible by a Nile Blue dye. Single catalyst particle mapping identifies minority species like the presence of a phosphated zeolite ZSM‐5‐containing FCC additive for selective propylene formation, catalyst particles without any zeolite phase and catalyst particles, which act as a trap for SO_x_.

## Graphical abstract

**From micro to macro**: A detailed correlation study between the metal content, acidity and accessibility of an industrial fluid catalytic cracking catalyst is presented, using laboratory‐based techniques that bridge the gap between single particle and bulk analyses. Single‐particle mapping revealed a large inter‐particle heterogeneity as well as the presence of minority species, including zeolite ZSM‐5‐containing catalyst particles.

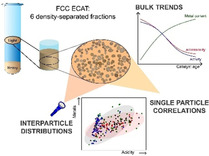

## Introduction

The main chemical process to produce gasoline and propylene is the cracking of vacuum gas oil (VGO) and heavy gas oil (HGO) with the use of a solid catalyst material. This multicomponent and hierarchically structured catalyst consists of spray‐dried porous spheres with an average diameter of 50–150 μm and contains next to zeolite also a clay and binder material, such as silica and alumina. The catalyst design is such that the mixture of hot catalyst and crude oil behaves like a liquid; hence the name fluid catalytic cracking (FCC).^1^, ^2^ The main active sites that catalyze the cracking of VGO are the Brønsted acid sites in the zeolite material, but the large crude oil molecules are pre‐cracked in the matrix of the FCC particle, prior to entering the crystalline micropores of the zeolite. Commonly employed zeolites in the FCC process include the synthetic ultra‐stable zeolite Y (ultra‐stable Y or US‐Y, FAU framework structure) and, to a lesser extent, zeolite ZSM‐5 with the MFI framework structure. Due to the smaller micropore structure of zeolite ZSM‐5 in comparison with that of zeolite US‐Y (i.e., 5.6 vs. 7.4 Å), zeolite ZSM‐5 increases the FCC catalyst selectivity towards propylene. They are added to the FCC catalyst inventory as separate FCC catalyst particles, and are visually not distinguishable from normal FCC catalyst particles containing zeolite US‐Y.^3^, ^4^

During the FCC process, VGO and HGO are cracked by the hot FCC catalyst in the riser reactor, after which the formed products are separated from the catalyst. Remaining carbonaceous species on the catalyst material block the active sites, preventing further cracking activity. The spent catalyst is, therefore, subjected to a regeneration procedure through high‐temperature calcination, making the FCC particles available for a consecutive cycle of crude oil cracking. The FCC particles can, however, also deactivate irreversibly due to the accumulation of metals originating from the crude oil feedstock, with Ni, Fe and V being the most notorious ones, resulting in a reduced porosity or metal promoted side reactions. Also, the harsh steaming conditions during catalyst regeneration induce dealumination of the embedded zeolite material, thereby decreasing the amount of available Brønsted acid sites. To preserve the overall activity in a reactor, part of the spent catalyst material is constantly being replaced with fresh FCC catalyst particles, leading to an equilibrium in the reactor with a mixture of FCC catalyst particles with different ages and degrees of deactivation. This mixture is called the equilibrium catalyst, further denoted as ECAT.^5^, ^6^

The previously mentioned deactivation processes, involving metal accumulation and zeolite dealumination, all occur simultaneously during the FCC process. Most studies, however, address only one of these processes, making use of lab‐deactivated FCC catalyst samples. Separating these two effects makes their investigation easier: the influence of dealumination due to steaming or the accumulation of metals coming from the oil feedstock on the catalytic performance of the FCC catalyst have been thoroughly studied on both the single particle and the bulk level.^7^, ^8^, ^9^ Furthermore, as mentioned before, due to the mix of fresh and deactivated FCC particles, the actual ECAT has a large intrinsic interparticle heterogeneity. This complicates the study of these industrially used catalyst particles even more.^10^

Our group has performed extensive work on the characterization of ECAT and FCC particles. Buurmans et al. used several staining techniques to visualize the acidity of ECAT and lab deactivated single FCC particles.^11^, ^12^ These staining techniques have been combined with for example TEM to correlate the acidity with structural features in one FCC particle.^10^ In later work by Ristanovic et al., extensive fluorescence microscopy work revealed more details on the activity of single zeolite domains by performing single molecule tracking.^13^ Kerssens et al. performed staining experiments on bigger samples of a few milligrams, to visualize the acidity of both fresh FCC and ECAT particles.^14^ For the accumulation of metals, Ruiz‐Martínez et al. revealed a core–shell deactivation due the accumulation of metals and coke using non‐invasive 2D micro X‐ray fluorescence (μ‐XRF). With X‐ray nano‐tomography, Meirer et al. studied the distribution of deactivating metals in more detail. They performed a density separation to classify three categories (low, medium and high metal loaded particles) and found that Fe forms a shell of reduced porosity in the outer 2 μm layer of the particle while Ni and V are distributed more throughout the whole particle.^15^ Most recently, the role of Ni in the deactivation of ECAT was studied with single particle X‐ray fluorescence‐diffraction‐absorption tomography by Gambino et al.^16^ Buurmans et al., however, already argued that a disadvantage of single particle characterization methods is the difficulty to obtain statistically relevant data.^8^ For example, due to the use of expensive and time‐consuming synchrotron related techniques,^17^ the amount of particles that can be analyzed is limited. Although the previously mentioned studies provided useful insights through extensive characterization of ECAT catalysts, they mainly focus on one deactivation process at the time with limited amounts of particles.

Recently, Solsona et al. did an individual assessment of multiple FCC particles via microfluidic sorting, based on the particles’ magnetic moment. The sorted samples obtained were too small to perform bulk characterization techniques, but it was possible to analyze a significant number of particles on the single particle level.^18^ For this analysis, after sorting the FCC particles, all collected fractions were analyzed with μ‐XRF mapping and fluorescence microscopy using staining molecules to characterize the fractions’ metal content, acidity and accessibility.

**Scheme 1:**
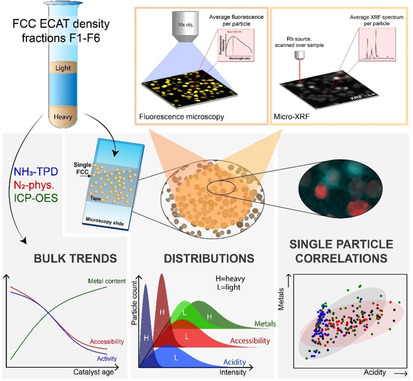
Schematic of the research approach. An FCC ECAT was density separated into fractions and analyzed for its acidity, accessibility and metal content. Bulk characterization techniques revealed trends as a function of density. Micro‐spectroscopic methods were employed for large scale single particle mapping. This screening allowed for determining interparticle distributions within each fraction as well as establishing single particle correlations.

Here, we continue this line of single particle diagnostics research by presenting a study about the influence of different deactivation processes on the activity of an ECAT. In particular, we report a characterization method, using laboratory‐based techniques, that bridges the gap between single particle and bulk analyses, as depicted in Scheme 1. We use the same set of characterization techniques as reported by Solsona et al. and try to further correlate the single particle mapping data on ECAT fractions sorted by a classical density separation. We have previously reported such a correlation to reveal interparticle heterogeneities in polymerization catalysts.^19^ We use these correlations to identify relationships as well as special FCC catalyst particles.

A calcined ECAT was sorted into six fractions, using density gradient separation with different diiodomethane/acetone mixtures.^20^ The increasing density per fraction is attributed to the accumulation of metals like Fe, Ni and V and, thus, to the catalytic age of a particle. It is generally accepted that the activity of an FCC particle decreases with increasing metal loading.^21^ By sorting this ECAT on density, the interparticle heterogeneity within each age fraction is reduced to facilitate a more precise characterization. Bulk characterization was established with traditional methods such as inductively coupled plasma‐optical emission spectroscopy (ICP‐OES), temperature programmed desorption with NH_3_ (NH_3_‐TPD) and N_2_ physisorption. In this work, the term “bulk technique” is used to address techniques that analyze a relatively large sample of ECAT particles and give an ensemble average as outcome. Each density separated ECAT fraction was, subsequently, characterized with multiple micro‐spectroscopic techniques to visualize the interparticle heterogeneity within each fraction. Specifically, fluorescence micro‐spectroscopy and subsequent μ‐XRF measurements were performed on a set of the same particles to obtain correlated information on many single FCC ECAT particles. Correlations between acidity, accessibility and metals accumulation could be made.

From this comprehensive approach, conclusions on three different levels could be drawn, as indicated in Scheme 1. First, bulk trends regarding the acidity, accessibility and metal accumulation as a function of catalyst age were established. Second, within each density separated fraction, large scale single particle mapping revealed the level of heterogeneity within these catalyst fractions. Finally, this particle screening resulted in detailed correlations regarding the catalyst properties and highlighted the presence of minority species that cannot be detected in neither bulk nor single particle analysis. The key of this presented methodology relies on single particle analysis with statistical relevance.

## Results and Discussion

**Figure 1:**
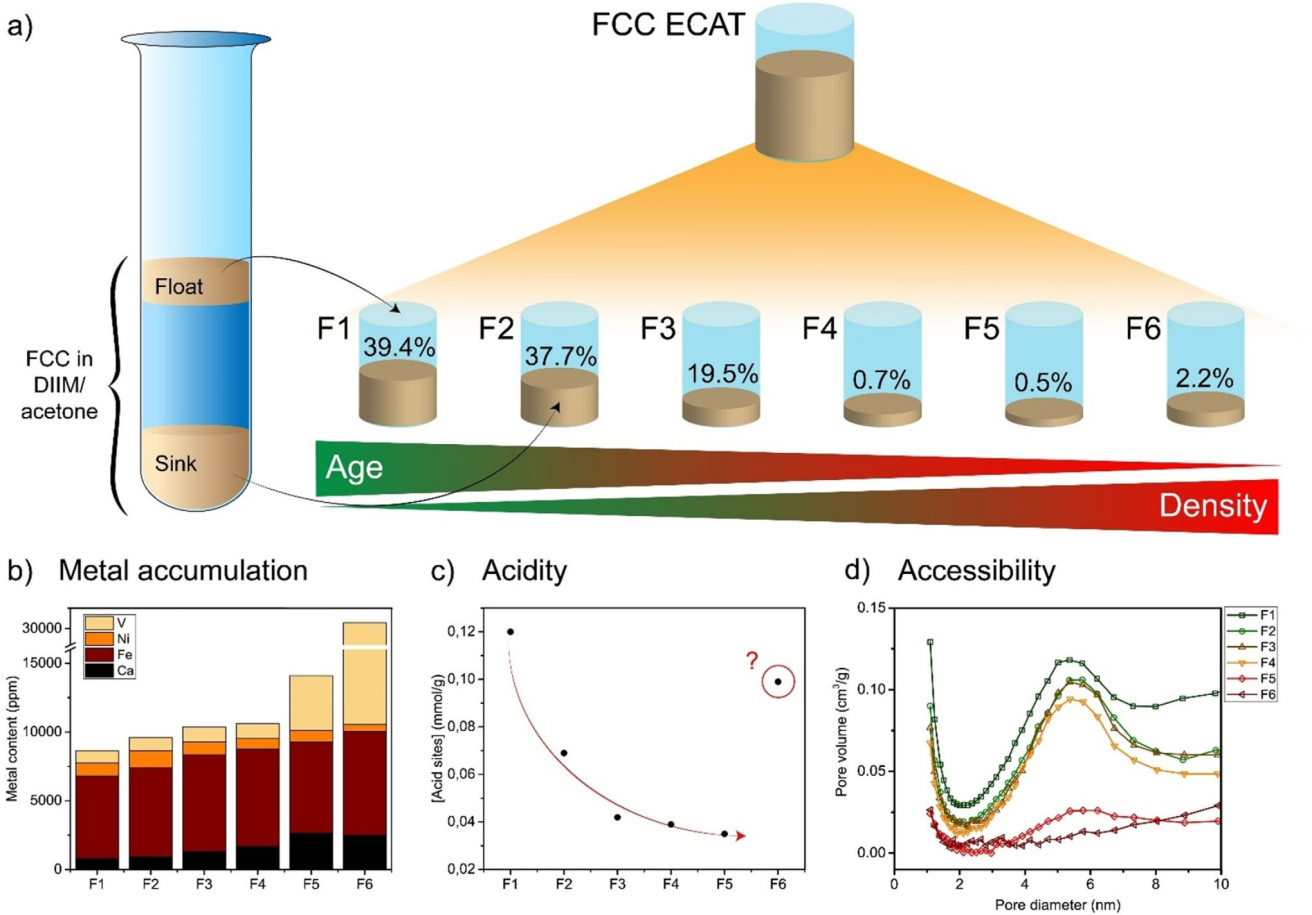
a) An FCC ECAT was density separated using a sink‐float method in different acetone/diiodomethane (DIIM) mixtures. The percentage of particles collected per fraction decreases with increasing density. Bulk characterization methods showed trends in metal accumulation, acidity and accessibility. ICP‐OES data (b) shows an increasing metal content. Quality control samples indicating the accuracy of the measurements give Fe=98 %, Ni=102 %, *V=*101 %, Ca=98 %. NH_3_‐TPD results (c) show a decreasing acidity and the pore size distribution as determined with N_2_ physisorption (d) shows a decrease in the amount of mesopores with increasing density. Fraction 6 does not follow these trends; this interesting outlier will be discussed in detail in the main text.

A regenerated ECAT from a commercial FCC unit was sorted in six fractions using a density gradient separation protocol, which was based on a sink‐float method in different acetone/diiodomethane (DIIM) mixtures. More details can be found in the Supporting Information. Figure 1 a and Table S2 demonstrate that most FCC catalyst particles end up in the fractions with the lowest density. More specifically, more than 60 % of the ECAT particles has a skeletal density below 2.47 g cm^−3^. In comparison, depending on the amount of zeolite material present and the source of binder material and clay, skeletal densities between 2.4 and 2.8 g cm^−3^ have been reported for fresh FCC catalyst particles.^21^−^23^

### Bulk catalyst characterization trends

Bulk characterization techniques, in this work referred to as techniques that were applied to a large sample of ECAT particles and including NH_3_‐TPD, N_2_‐physisorption, and ICP‐OES, revealed the bulk trends as function of density in acidity, accessibility and metal accumulation, respectively. Together, these bulk analyses provide useful insights into the level of catalyst deactivation as a function of catalytic age. In correspondence with previous studies, the metal content of FCC particles is correlated with density, while acidity and accessibility are negatively correlated with density. Since we are investigating an industrially used and regenerated ECAT, all particles already have been used in at least one cracking cycle and could, theoretically, already be considerably deactivated. Therefore, we expect a relatively low overall acidity and accessibility in the particles.

Figure 1 b indicates increasing levels of Ca, Fe, and V with increasing density, suggesting longer residence times in the FCC reactor. Also, the overall level of Ni is relatively low in this specific FCC ECAT.

Three striking observations can be made from these bulk trends. First, as seen in Figure 1 c, the decrease in acidity appears most significant going from fraction F1 to F2 and from F2 to F3, while fractions F3 to F5 have similar acidities. CO FT‐IR spectroscopy (Figure S4) indeed confirms that most Brønsted acid sites are removed going from fraction F1 to F2 and indicate that strong Lewis acid sites even only exist in fraction F1. Also, mesopores of around 6 nm (Figure 1 d) and the micropore volume (Figure S2c) decrease significantly from fraction F1 to F4. This is a clear indication of the destruction of zeolite domains, the only microporous material in an FCC particle, during the aging of the particle. Furthermore, the mesopores of 20–30 nm as observed in fraction F1 (Figure S2b), disappear completely upon ageing. This is ascribed to the accumulation of metals in the matrix of the particles, blocking the accessibility for oil molecules.

Second, both fractions F5 and F6 appear to lack a presence of zeolite domains, as both fractions demonstrate a low level of La compared to fractions F1–4 (see ICP‐OES results in Figure S1). The La level can be used as a marker for zeolite US‐Y domains, since this rare earth metal is used as stabilizing agent during the synthesis of this zeolite.^24^, ^25^ Therefore, fraction F5 and F6 contain non‐FCC particles without zeolite domains. Considering only fractions comprising actual FCC particles (fraction F1 to F4), their La levels are fairly similar for fractions F1 to F3, which is an indication for a comparable amount of zeolite domains in these fractions, and slowly decreases to fraction F4. From literature, it is not expected that La will leave the particles upon zeolite framework collapse.^25^ The absence of zeolite domains in fractions F5 and F6 is further rationalized by the deviating pore size distribution as indicated in Figure 1 d. These two observations indicate that employing density gradient separation allows for filtering out most non‐FCC particles from the ECAT batch.

Third, the composition of fraction F6, the heaviest fraction, does not follow the trends set by the first five fractions. The Mg and V levels are particularly high in this most dense FCC fraction, while as shown in Figure S1 the level of Ti, a marker for the clay component of a typical FCC particles’ matrix^26^ is very low. This suggests that fraction F6 mainly contains Mg‐based traps for vanadium or SO_x_,^3^, ^27^, ^28^ which we were able to separate from the FCC catalyst particles using density separation. These particles possess a low porosity, but have a relatively high Lewis acidity, most probably due to the presence of acidic vanadium oxide.

### Particle distributions within density‐separated catalyst

The bulk characterization techniques as discussed in Figure 1 provide overall trends and insights in the investigated ECAT material. Single‐particle mapping, however, provides a more in‐depth analysis of the interparticle heterogeneity of the investigated FCC samples. These results give distributions of the acidity, accessibility and metal accumulation in every fraction. For every fraction, a minimum of 90 particles was analyzed. The exact amounts are given in Table S3.

**Figure 2:**
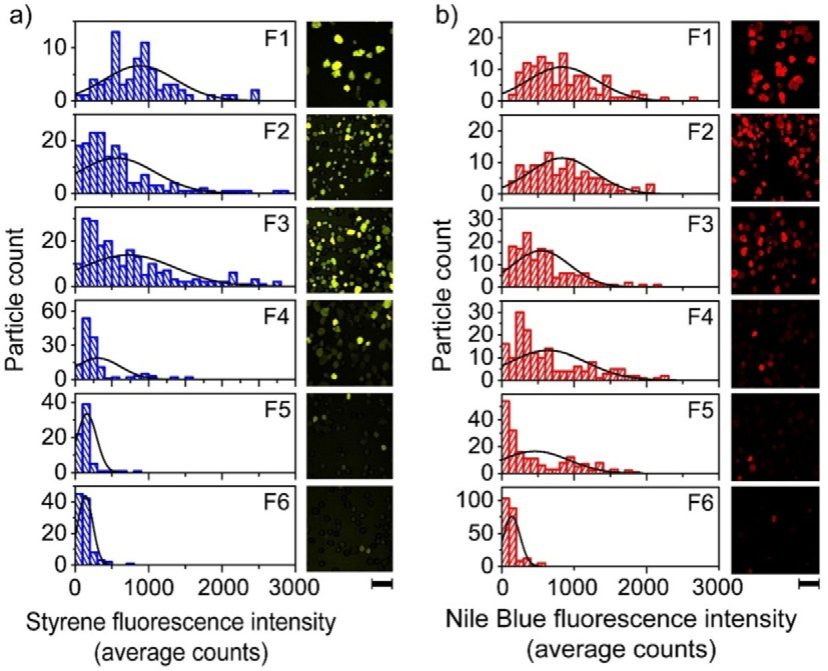
Single particle mapping revealing interparticle distributions within each density separated ECAT fraction. For each particle in field of view, the fluorescence of styrene oligomerization reaction products indicates the level of acidity (a) and the fluorescence of Nile Blue demonstrates the accessibility (b). Each scale bar equals to 200 μm.

Figure 2 a shows histograms of the fluorescence intensity per particle after the Brønsted acid catalyzed oligomerization of 4‐methoxystyrene. This fluorescence intensity is a measure for the number of available acid sites. With this staining method, using a styrene derivative as probe molecule, the amount of available acid sites can be visualized, as was shown before by Buurmans et al.^8^, ^11^, ^12^ In line with our previously shown bulk results in Figure 1 c, the acidity decreases with density. For an assessment of the particles’ accessibility, staining experiments were performed using Nile Blue A, a large conjugated fluorescent molecule that can enter the macro‐ and mesoporous pore structure of an FCC particle.^8^ Similar to the acidity measurements in Figure 2 a, the fluorescence intensity from Nile Blue reflects the particle accessibility. The distributions in Figure 2 b indicate that also the average accessibility decreases with increasing density.

Strikingly, the trends in the acidity distributions show an almost bimodal behavior. Within the fractions F1–F3, the distributions of acidity demonstrate a large spread and thus significant interparticle heterogeneities. Fractions F4–F6, however, show more narrow distributions with low fluorescent intensities. We ascribe the heterogeneity in the lighter fractions to a convolution of a natural density spread and ageing. Due to the spray drying process, we expect a density gradient already in the fresh catalyst. Particles that are, therefore, denser from the start, can end up in a fraction together with aged particles. Fraction F4 only shows non‐fluorescent particles, suggesting the absence of acid sites and the blockage of pores. The decrease in porosity is less pronounced than the drop in acidity. The large spread in the accessibility of particles in Fraction F4 suggests that the deactivation of Fraction F4 is due to a combination of pore blocking by Fe, as reported by Meirer et al. and the absence of acid sites.^21^, ^29^

A different explanation for the non‐fluorescent particles in all fractions, relies on the presence of other type of particles, such as additives or spray‐dried particles without zeolites. The absence of Brønsted acid sites can be a result of synthesis or due to severe dealumination. Bulk analysis showed that such particles are typically found in fractions F5 and F6. Regardless of the origin, these so‐called non‐FCC particles are not as porous as the active FCC particles and do not contain Brønsted acid sites to perform the oligomerization of 4‐methoxystyrene. Due to a low metal loading, however, they can also end up in the lightest fractions. Although they show low fluorescence intensities regarding acidity and accessibility, they are properly sorted into the correct age fraction.

**Figure 3:**
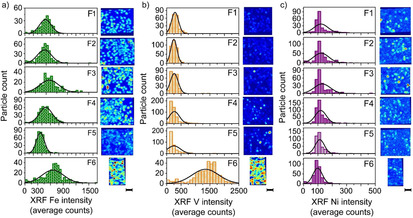
Single particle mapping revealing interparticle distributions within each density separated ECAT fraction. For each particle in field of view, the XRF intensity represents the content of Fe (a), V (b) and Ni (c). Each scale bar equals to 200 μm.

Finally, μ‐XRF experiments provided the XRF intensity of different metals per particle. In Figure 3, the distributions of Fe, V and Ni as a function of density are shown. The average μ‐XRF values per element and fraction follow the trends as previously observed with bulk analysis (ICP‐OES). Figure S5 presents the average μ‐XRF values for the other most abundant metals as indicated with ICP‐OES. It was found that for each metal, the average μ‐XRF intensity per density separated fraction follows the trend from bulk analysis. This means that the single particle mapping method provides an accurate representation of the bulk material, indicating that we have studied a statistically relevant number of particles. The main advantage of this μ‐XRF mapping over bulk methods like ICP‐OES, however, is that interparticle heterogeneity is revealed.

The Fe distributions appear narrower compared to styrene and Nile Blue in Figure 2. Also, the differences between fractions are less pronounced and more difficult to visualize. Therefore, to show clear trends and capture outliers with either very low or high Fe contents, we need more particles as compared to the acidity and accessibility studies. It is interesting to see the broad V distribution of fraction F6, while the other fractions contain only limited amounts of V. The distributions of Ni are narrow and at relatively low intensities and indicate an overall low amount of Ni in this ECAT. All μ‐XRF images per fraction are of the same region and can therefore be directly overlaid.

### Correlations between metal type and content and catalyst particle acidity and accessibility

By performing μ‐XRF and fluorescence microscopy measurements at identical positions, single particle mapping can also provide correlations between the acidic properties and the metal content of particles. These correlations allow for detecting the presence of minority species, provide additional information about the FCC ECAT, or reveal new trends that were not revealed with bulk analyses.

In FCC deactivation research, the Ni content of a particle is often used as indication for the age of a catalyst: Ni is accumulated during the cracking process but is not present in the fresh catalyst (such as Fe in the clay component of the FCC catalyst). Furthermore, Ni inside an FCC particle is not very mobile. Therefore, the more Ni an ECAT particle contains, the longer it has been cycling through the reactor and the more Fe and V it contains.^30^ Consequently, it is expected that these particles show a low acidity and accessibility and thus a low fluorescence of Nile Blue (NB) and 4‐methoxystyrene.

**Figure 4:**
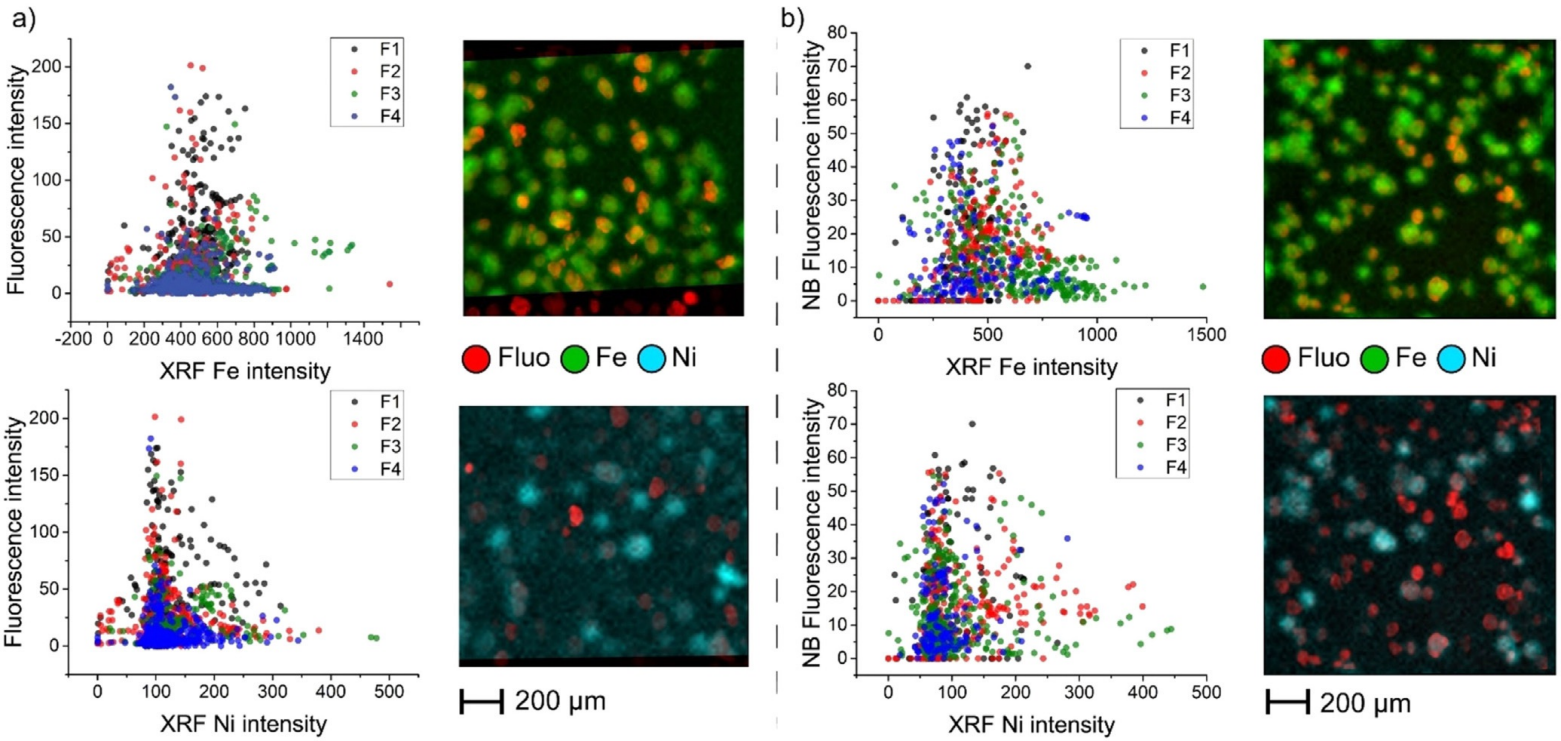
The correlation plots of Fe (green in image) and Ni (blue in image) with fluorescence of 4‐methoxystyrene (a) and Nile blue (b) with corresponding example images show the relation between deactivating metals and acidity/accessibility. Pearson correlations of these plots are −0.014, 0.0011, −0.11 and 0.049, indicating there is no linear correlation found in all plots.

Correlation plots show the relation between fluorescence, Fe and Ni, where each measured particle is represented with one data point. These plots originate from the overlay of the corresponding μ‐XRF maps and the resulting single particle intensities. A detailed explanation of the data analysis can be found in Figure S6. The overlaying maps of acidity and Ni in Figure 4 a demonstrate an anti‐correlation between acidity and Ni content in each fraction. Closer inspection reveals that particles with a high Ni content always show low fluorescence, and therefore low acidity. We can therefore conclude that indeed, Ni acts as a marker for catalytic age of an ECAT. It must be noted that a low level of acidity is indeed linked to a higher degree of deactivation and not to an absence of zeolite domains, as indicated in Figure S7. Due to the overall low levels of Ni present in this ECAT, bulk characterization techniques were not sufficiently accurate to detect this new trend. The correlation of Ni with NB staining (Figure 4 b) is less pronounced, but also here particles with a high Ni content show low accessibility. The correlation between Fe and acidity or accessibility is not as straightforward. We expect a relation where acidity and accessibility decrease with increasing Fe level, due to pore blocking. However, the overlay images in Figure 4 show particles with a high Fe content that still show 4‐methoxystyrene (left) or Nile Blue (right) fluorescence. In these particles, Fe is most likely deposited as clusters. These Fe clusters were already observed in the study of Solsona et al., in which it was demonstrated that particles with high Fe levels are not necessarily heavily deactivated.^18^

**Figure 5:**
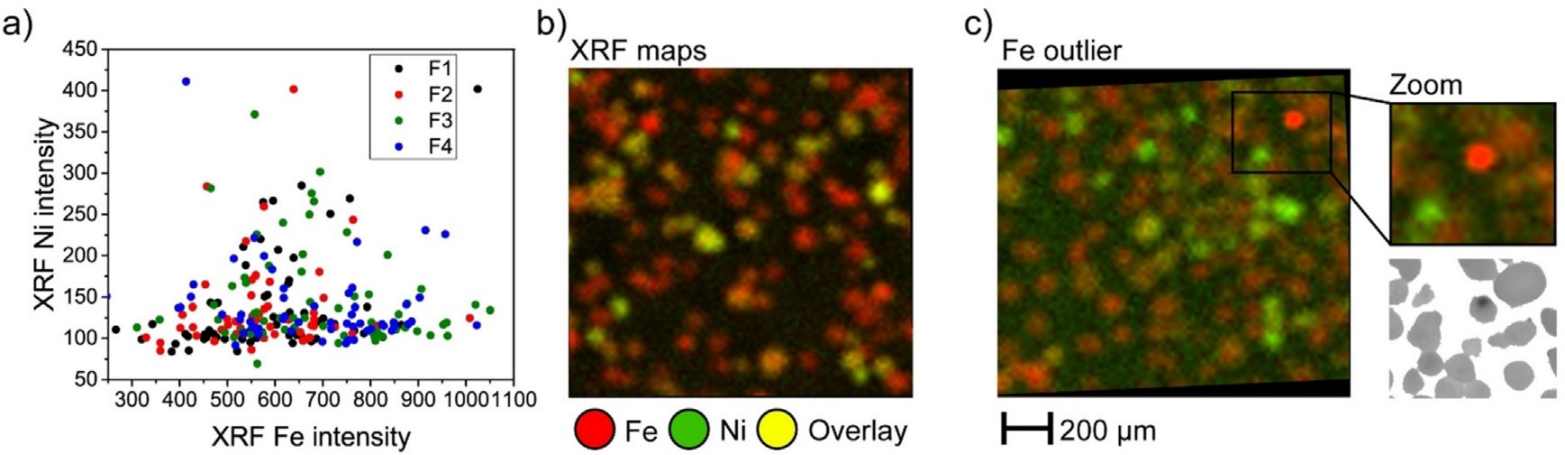
The correlation plot of the XRF maps of Ni and Fe (a, Pearson correlation: 0.26). Every data point represents an individual particle with Pearson correlation coefficients of 0.54, 0.16, −0.12, −0.18 for F1 to F4 respectively. The overlay images (b) of fraction 2 are shown as an example. The second example (c) shows a Fe outlier with a very high XRF intensity, attributed to a cluster. The threshold of the overview Fe image is chosen as such to display the overall image correct; the outlier is therefore saturated. See Figure S9 (Supporting Information) for details.

The different correlations between Fe and Ni with acidity and accessibility, lead to the question how Fe and Ni relate to each other. Figure 5 a shows the Ni–Fe correlation plots as a result of overlaying the single particle XRF maps. The correlation appears to be a fan‐shaped plot, indicating that particles with high Ni concentrations generally also have elevated Fe levels. The reverse correlation, on the other hand, does not uphold, meaning that particles with a high Fe concentration do not necessarily have a high Ni content as well. The hotspot of Fe that is observed in the upper right corner of Figure 5 b, is allocated to an FCC particle with a dark spot on the optical image in Figure 5 c. This particle has, thus, a cluster with high concentrations of Fe, but overall, does not have a high skeletal density, as it was sorted in fraction F2. Interestingly, whereas bulk characterization demonstrated that the Fe and Ni contents gradually increase with increasing density, single particle mapping now indicates a significant heterogeneous interparticle distribution.

### Minority species

**Figure 6:**
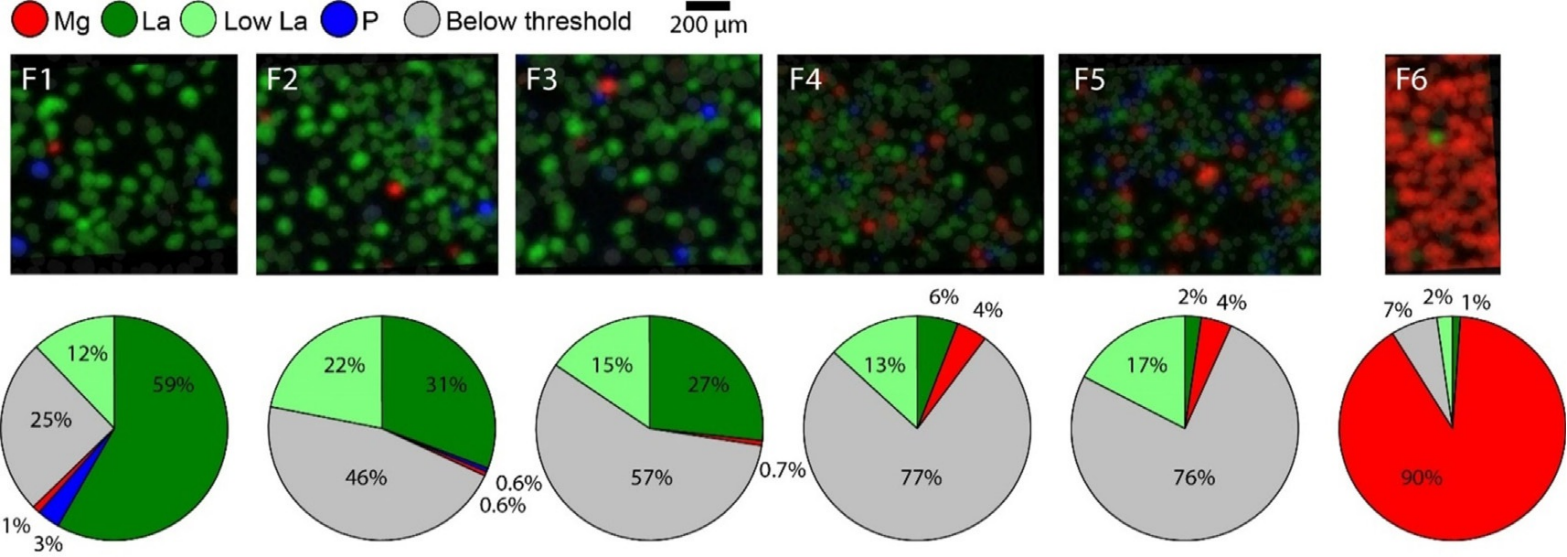
Overlay of XRF maps Mg, La and P for fraction F1–F6 and relative percentages of all three. Particles with an XRF intensity for La between 200 and 600 are indicated as light green in the pie charts. More details of this analysis can be found in Figure S9. The blue colour in the overlay image of fraction F5 is below the set threshold and therefore not ascribed to ZSM‐5 domains, but to other additives. The pie charts represent the percentages of the number of particles with Mg, La or P as main content.

**Figure 7:**
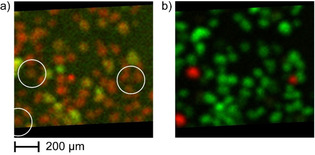
(a) An overlay of Ni (green) and Fe (red) in fraction F1 shows that the zeolite ZSM‐5‐containing catalyst particles (within white circles) generally contain below average levels of Fe and/or Ni. In (b) the ZSM‐5 particles are indicated red (P) while USY particles are indicated green (La).

Our presented single particle mapping method provides the possibility to determine the composition of many single particles within each fraction. This allows for the detection of outliers and minority species. As was stated before, FCC particles can contain both ZSM‐5 and US‐Y as active cracking components. Like La serves as a marker for zeolite US‐Y, the presence of ZSM‐5 in a particle is indicated by the P content. The ICP‐OES measurements in Figure 1 b did not detect a significant amount or clear trend in the P content per fraction. On the single particle level, however, we observe the presence of ZSM‐5 containing particles in fractions F1 and F2. Figure 6 shows these particles in blue. Interestingly, as shown in Figure 7, these particles contain low levels of Fe and Ni. Second, the Mg based traps for V/SO_x_ are more clearly detected with XRF mapping. Detailed correlation plots in Figure S8 show the direct link of Mg with V. The red indicated particles (Mg) in Figure 6 demonstrate that minor amounts of these traps were collected in fractions F1 to F5, but the majority is collected in fraction F6. Particles not displaying one of the pure distinct colors red, blue, or green, do not contain (enough) zeolite domains (green, blue) or Mg (red) and are indicated as grey in the pie charts. It is important to note that these thresholds are chosen manually; a detailed description can be found in the Supporting Information. From the pie charts, once more the large intrinsic heterogeneity of an FCC ECAT is emphasized. Most striking from these results is the heterogeneity of the La values found for all particles. The large spread in zeolite content is most probably due to the used spray drying technique and again highlights the importance of a method that can perform diagnostics on many single particles: bulk methods cannot give enough information on a sample as complex as an FCC ECAT material. This characterization method can be extended to other systems with a large inter‐particle heterogeneous catalysts, such as olefin polymerization catalysts or metal (oxide) impregnated support oxides.^19^, ^31^

## Conclusions

We have shown that correlating information from multiple techniques per particle, provides valuable insights in the heterogeneity of a density separated FCC ECAT. Furthermore, the possibility to measure multiple particles adds statistical value to the data obtained. Where bulk measurements can give insight in the trends between the six density separated FCC ECAT fractions under study, the diagnostics of single particles can show trends even within each fraction.

Extensive characterization revealed clear trends in the acidity, accessibility and metal content of the density separated fractions: from fractions F1 to F4 the separation was according to catalytic age. We conclude this from the accumulation of deactivating metals, a decrease in acidity and accessibility. However, we have shown a large spread in acidity and accessibility for these fractions as a result of a convolution of natural density and ageing. Fractions F5 and F6 are mainly composed of additives without zeolites or Mg‐based traps for V or SO_x_.

The large intrinsic inter‐particle heterogeneity of each density separated fraction was visualized by overlaying XRF maps of Fe and Ni with fluorescence maps. The degree of deactivation was linked to the Ni content on a single particle level, in a sense that particles with a high Ni content do not show high acidity. Furthermore, correlations of the Mg, La and P levels revealed the presence of ZSM‐5 containing particles in the lightest fractions, while the V/SO_x_‐traps were collected with increasing amounts in the heavier fractions.

## Experimental Section

A detailed experimental approach of the described work can be found in section S1 of the Supporting Information (SI). A regenerated ECAT sample, originating from a commercial FCC unit, was calcined at 600 °C for 5 h to remove residual coke species. Subsequently, density gradient separation, as described by Dyrkacz et al.,^32^ was used to sort the ECAT catalyst particles in six fractions with increasing density. The density separated catalyst fractions have been characterised by Temperature Programmed Desorption (TPD) of NH_3_ using a Micromeritics ASAP2920 equipped with a TCD detector, Fourier Transform‐Infrared Spectroscopy (FT‐IR) spectroscopy in transmission mode and CO as probe molecule using a PerkinElmer 2000 instrument, N_2_‐physisorption using a Micromeritics TriStar apparatus and Inductively Coupled Plasma–Optical Emission Spectroscopy (ICP‐OES) measurements using a Spectro Arcos instrument. For the single catalyst FCC particle studies, micro‐X‐ray Fluorescence (μ‐XRF) studies were performed with an Orbis PC SDD instrument with a Rh tube as X‐ray source, while the fluorescence microscopy studies in combination with probe molecules were done using a Nikon upright A1 confocal fluorescence microscope equipped with a 488 nm excitation solid‐state laser source. The probe molecules were 4‐methoxystyrene (Sigma Aldrich, 97 %) and Nile Blue A (Acros Organic, pure). Data correlations were made using an in‐house MATLAB script to register fluorescence and μ‐XRF maps with an optical image for every density separated catalyst fraction.

## Conflict of interest

The authors declare no conflict of interest.

## Supporting information

As a service to our authors and readers, this journal provides supporting information supplied by the authors. Such materials are peer reviewed and may be re‐organized for online delivery, but are not copy‐edited or typeset. Technical support issues arising from supporting information (other than missing files) should be addressed to the authors.

Supplementary
