# Supplementary material for: Single Particle Assays to Determine Heterogeneities within Fluid Catalytic Cracking Catalysts
Source: Chemistry. 2020 May 29;26(39):8546–54. doi: 10.1002/chem.201905880 (PMC7384009; doi:10.1002/chem.201905880)
Supplement: Supplementary file 1 — Supplementary [file CHEM-26-8546-s001.pdf]

Chemistry–A European Journal

Supporting Information

Single Particle Essays to Determine Heterogeneities within Fluid Catalytic Cracking Catalysts

Anne-Eva Nieuwelink+, Marjolein E. Z. Velthoen+, Yoni C. M. Nederstigt, Kristel L. Jagtenberg, Florian Meirer, and Bert M. Weckhuysen*[a]

Supporting information

Section S1: Experimental

Sample preparation: A regenerated FCC ECAT, obtained from an industrial FCC unit was dried at 120 °C for 1 h (ramp: 1 °C/min), followed by calcination at 600 °C for 5 h (5 °C/min) to remove residual coke species. The slow drying process was performed to remove H_2O from the FCC particles without further steaming and hence dealumination of the zeolite domains, leading to no or minimal further deactivation.[1–3] Subsequently, density gradient separation as described by Dyrkacz *et al* [4] was used to sort the FCC ECAT particles in six fractions with increasing density. For this purpose, diiodomethane (DIIM, Sigma-Aldrich, purity 99%)/acetone (GPR RECTAPUR, VWR Chemicals, purity >99.5%) mixtures with different ratios were prepared as listed in Table S1. For the first sorting step, 2.5 g FCC ECAT particles were added to a 20 cm tube (Ø=1 cm) and dispersed in 15 mL of solution 1. After 2 h, the particles were equilibrated and two layers were created: a heavy fraction at the bottom of the tube and a floating fraction on top of the DIIM/acetone solution. The upper layer was removed, washed with acetone and dried in air. This fraction is herein referred to as Fraction 1. The lower layer was re-dispersed in 15 mL of DIIM/acetone solution 2 to split off a second, slightly denser, fraction that was treated in a similar manner as Fraction 1 and is herein referred to as Fraction 2. This process was repeated with solutions 3 to 5. After the last step, also the bottom layer was washed and dried. All six fractions were calcined following the same procedure as described above to remove residual solvent. In this way the FCC ECAT was separated into six fractions with increasing density, herein denoted as Fractions 1-6.

Table S1: Solutions used for density gradient separation of calcined FCC ECAT particles.

	**DIIM:acetone (%vol)**	**Calculated density (cm^3/g)**
**Solution 1**	1.5:1	2.31
**Solution 2**	2:1	2.47
**Solution 3**	2.5:1	2.60
**Solution 4**	3:1	2.64
**Solution 5**	5:1	2.90

Bulk Characterization: Temperature programmed desorption (TPD) of ammonia (NH_3) measurements were performed using a Micromeritics ASAP2920 equipped with a TCD detector. Typically, 0.1 g of sample was dried *in situ* under an He flow at 550 °C for 30 min (ramp 10 °C/min). The sample was subsequently cooled down to 100 °C. At this temperature, NH_3 pulses of 25.17 cm^3/min were applied until saturation of all acid sites. The sample was then heated to 550 °C (ramp 10 °C/min) to induce desorption of NH_3.
Fourier Transform Infrared (FT-IR) spectra were recorded in transmission mode on a Perkin-Elmer 2000 instrument employed with a DTGS detector using 32 scans per spectrum and a resolution of 4 cm^{-1}. Typically, 0.015 g of sample were pressed into self-supported wafers (Ø=12 mm) and placed in a cell that allows switching between vacuum and CO gas. Samples were dried *in situ* at 550 °C for 30 min (ramp 10 °C/min) under a dynamic vacuum. CO (10% in He) was dosed at low temperatures (-188 °C) and at low pressures (between 1.0 and $1.5 \cdot 10^{-3}$ mbar) with spectra being recorded after each pulse.
NH_3-TPD provides an indication of the overall acidic properties and an accurate quantification of the total amount of acid sites, while CO FT-IR spectroscopy provides more

insight into the nature and strength of the different acid sites present in the studied samples.
The average accessibility of FCC catalyst particles as a function of age was determined with N_2-physisorption. N_2 physisorption was performed at -196 °C using a Micromeritics TriStar apparatus. Samples were dried using N_2. The mesopore volumes (2 – 300 nm range) and Barrett –Joyner–Halenda (BJH) pore size distributions were determined using the adsorption branch of the isotherm with Aerosil 380 as a reference. Micropore volume was determined with the tPlot using the linear part of the adsorption branch between 0.275 and 0.3 nm (statistical thickness) with Aerosil 380 as a reference.
With a quantitative Inductively Coupled Plasma Optical Emission Spectroscopy (ICP-OES) measurement, nature and abundance of all metals present in the FCC ECAT fractions can be determined. ICP-OES measurements were performed using the SPECTRO ARCOS ICP-OES spectrometer. Typically, 100-150 mg of sample was dissolved in HF and HNO_3 to remove the Si/Al framework of the catalyst. The samples were measured in an emission range of 125-770 nm.

Single Particle Characterization: We have studied the accessibility and acidity of single FCC particles with staining experiments using fluorescence microscopy. It is known from previous research in our group, that staining methods using probe molecules like thiophene, styrene or furfuryl alcohol can be used to study the acidity of zeolites. These staining methods are based on a Brønsted acid-catalyzed oligomerization reaction of the probe molecules. Upon oligomerization, conjugated reaction products are formed that can be detected with UV-Vis, optical or fluorescence spectroscopy.[5–7] 4-Methoxystyrene was selected here due to its relatively high reaction rate constant: the methoxy group stabilizes the carbocation formed via the formation of resonance structures.[8] This makes 4-methoxystyrene also active towards oligomerization on weaker acid sites. In contrast, 4-fluorostyrene can only oligomerize on the very acidic Brønsted acid sites of ZSM-5 and is, therefore, more selective, but less relevant for this study. Additionally, a non-reactive bulky molecule like Nile blue A can be used to probe the accessibility of the FCC particles' pore structure. Nile blue A cannot enter the zeolite pore structure, but it can enter the mesoporous and macroporous structure of the matrix. Therefore, the fluorescence of the FCC particles caused by Nile Blue A can be taken as a measure for the catalyst's accessibility for VGO molecules.[5,7,9–11]
4-Methoxystyrene and Nile Blue A show fluorescence in the same wavelength regions. Therefore, the experiments with Nile Blue A to probe the accessibility of the density separated FCC ECAT fractions have been performed on a new sample from the same fraction. The sample was immersed in a 0.1 mM Nile blue A solution in ethanol. After an equilibration time, the fluorescence intensity per particle was measured. We expect that the uptake of Nile blue A by an FCC particle is dependent on the amount of accessible pores. Therefore, we use the fluorescence intensity of an FCC particle after Nile blue A staining as a measure for accessibility of the particles' matrix.
Each exact same sample was also analysed employing micro X-ray fluorescence (XRF) mapping to measure the metal content per particle. Micro-XRF spectroscopy is a spectro-chemical tool for determining in a spatially resolved way the composition of (typically inorganic) materials. Upon excitation with a focused X-ray beam, characteristic X-rays are emitted by the sample. The detected XRF spectra can be fitted to identify the elements present in a material, with a spatial resolution that is determined by the size of the X-ray beam.[12]
The micro-XRF method in this study was only used to measure relative intensities. The reference material, for example a fresh FCC catalyst from the same reactor unit, required

to perform a correct calibration, was not available. However, these results give valuable information about the trends within the different particles from the density separated FCC ECAT fractions.

For these measurements, each fraction was placed on tape to create a surface covered with particles. Two duplicates were prepared and placed in the chamber of a micro-XRF Orbis PC SDD with a Rh tube as X-ray source. An XRF map was collected with 30 μm resolution and a 15 μm step size. Next, using a homemade MATLAB script, the maps were segmented to combine the spectra per particle into one average spectrum for every particle. This allowed for the analysis of ±100 individual FCC particles per fraction. Consecutively, one of the XRF samples was stained with Nile Blue A and the other with 4-methoxystyrene. The Nile Blue A staining was performed by immersing the particles in a 0.1 mM solution of Nile Blue A and equilibrated for 1 h. 4-Methoxystyrene was added via diffusion by placing a 10 μL droplet next to the sample. In a closed environment, the sample was heated treated at 100 °C for 15 min. Both samples were illuminated with a Nikon upright A1 confocal fluorescence microscope equipped with a 488 nm excitation solid-state laser source. Images were collected using a 10x objective to fit ±80 FCC particles in one image.

The fluorescence images were complemented with an optical image of the same region. These images were collected using a Zeiss Plan Neo Fluar upright microscope. With a second in-house designed MATLAB script, the fluorescence and optical images and the XRF maps were digitally resampled to the same pixel size and then registered to overlay all information of each individual particle. This registration step resulted in a multi-dimensional image where each pixel contained information from all used imaging methods. This allowed generating correlation plots of all present metals, the fluorescence intensity, and the observed color of the particles.

Section S2: bulk characterization of density separated fractions

Table S2: Weight and average density of FCC ECAT fractions obtained after density gradient separation in DIIM/Acetone mixtures. Density of mixture is calculated based on ratio of DIIM and acetone; it is assumed that these liquids are fully miscible. Percentages are based on total collected amount. For details, see Experimental section.

Fraction	amount (%)	density (g/cm^3)
**1**	39.4	<2.31
**2**	37.7	2.31-2.47
**3**	19.5	2.47-2.60
**4**	0.66	2.60-2.64
**5**	0.46	2.64-2.90
**6**	2.22	>2.90

Figure 1b in the manuscript indicate the metal content as a function of FCC particle density, as determined with ICP-OES. These results demonstrate that density gradient separation of an FCC ECAT indeed leads to fractions with increasing loading of deactivating metals and, therefore, with increasing age. Figure S1 provides a more detailed sub-division in metals that can be used as markers and metals coming from the VGO that accumulate in the particles during catalysis.

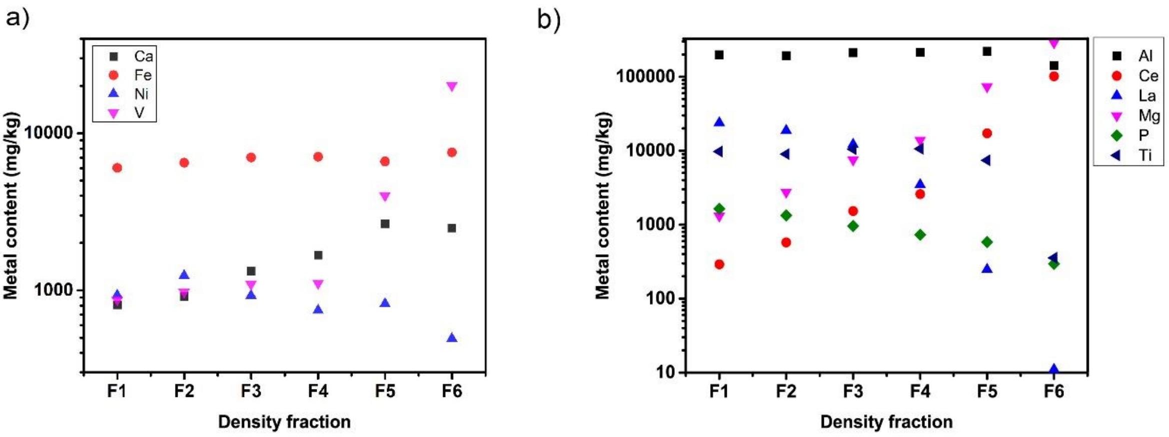

Figure S1: ICP (please note the logarithmic scale) data of most important deactivating metals (a) and elements that can be used as markers (b) for specific domains within the particles in the six density separated FCC fractions.

N_2 physisorption was employed to determine the accessibility properties of each fraction under study. Figure 1d in the manuscript presents the mesopore size distribution for all fractions. Figure S2 also shows the N_2 physisorption isotherms (a), the pore size distribution for all fractions over the whole mesopore range (b) and the micropore volume (c). Fractions 1-4 express similar pore size distributions with considerable micropore volumes and a significant amount of small mesopores (6 nm), which are both inherent to the zeolite domains. Fraction 5, on the other hand, lacks these features and demonstrates a broad distribution of larger mesopores, ascribed to the matrix of FCC particles, confirming the absence of zeolite domains. Fraction 6 has a completely different pore size distribution, containing mainly pores of around 50 nm. These observations are in agreement with ICP-OES results, establishing the fact that fractions 1-4 contain FCC particles with increasing age, fraction 5 contains the so-called filler particles without zeolite domains and fraction 6 mainly consists of V traps.

Comparing fractions F1-4, it can be observed that the micropore volume decreases significantly with a factor 2 going from fraction 1 to 2 and then slowly decreases further with increasing density to fraction 4. This crystallinity loss is ascribed to the dealumination and consequent structural collapse of the zeolite framework by steam during the regeneration of the FCC particles. The zeolite mesopores are more resistant against ageing.

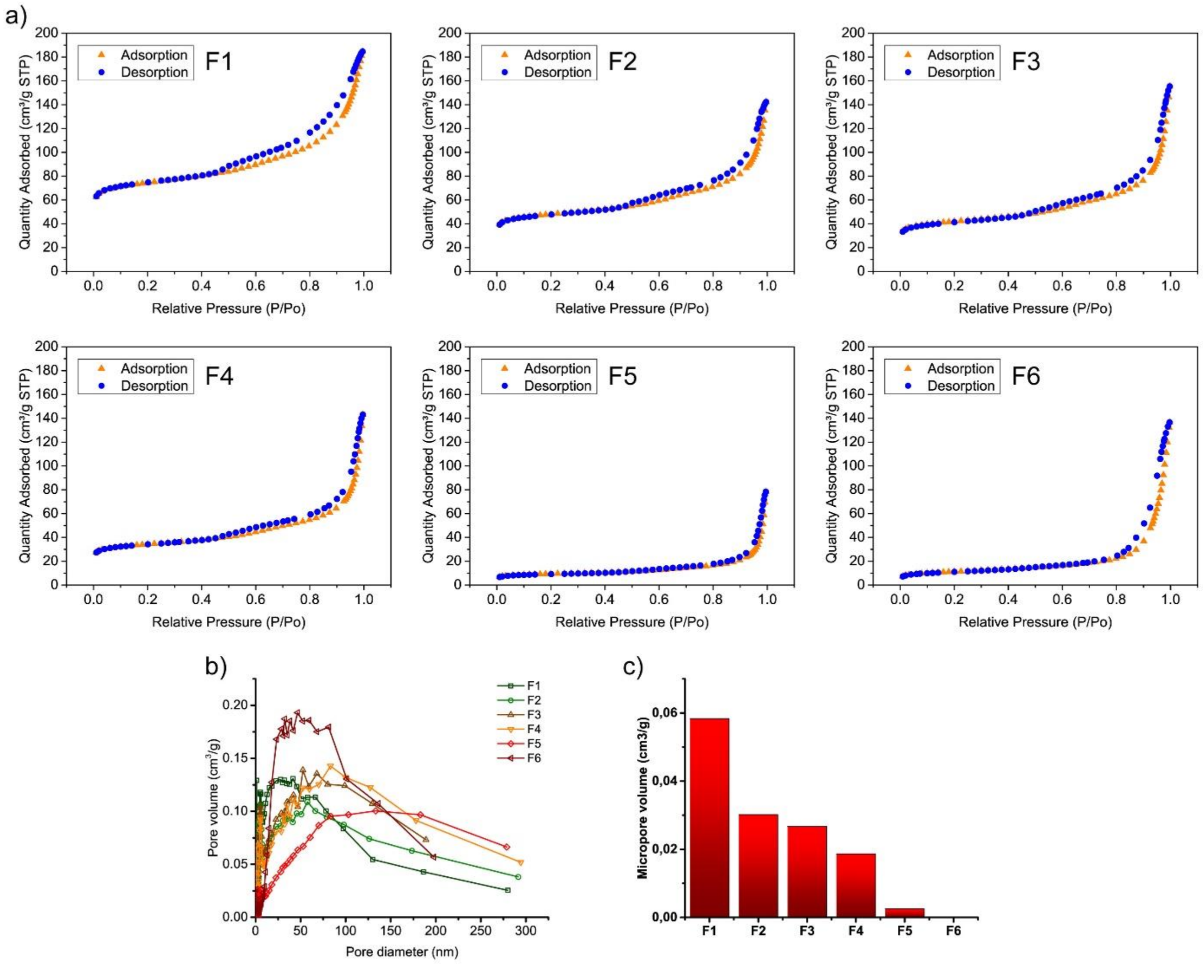

Figure S2: N_2 physisorption isotherms (a), pore size distribution for all fractions over the whole mesopore range (b) and micropore volume (c).

The activity of an FCC particle in the cracking reaction depends on the presence of acid sites accessible to oil molecules, both in the matrix and in the zeolite domains. The matrix contains Lewis acid sites, capable of pre-cracking the large oil molecules and the zeolite domains possess Brønsted acid sites to crack the molecules into the desired products. Particles with a higher degree of dealumination and metal accumulation are, therefore, expected to possess a lower amount of acid sites and, by extension, expected to be less active. To support this hypothesis, the acidic properties of the six fractions under study were determined and taken as a measure for activity. NH_3-TPD characterization resulted in a quantified amount of acid sites per gram, as indicated in Figure 1c in the manuscript. The corresponding TPD profiles can be found in Figure S3 in the Supporting Information. There is a clear decrease in the amount of acid sites per gram with increasing density. Going from fraction 1 to 2, the amount of acid sites decreases significantly, similar to what was observed for the accessibility with N_2 physisorption. Then, the amount of acid sites gradually decreases further during the ageing of FCC particles (fractions 1-4). The acidity of fraction 5 is low, due to the absence of zeolite domains. To obtain more insights into the nature of the different acid sites present in the different fractions, FT-IR spectroscopy with CO as a probe molecule for acid sites was employed.

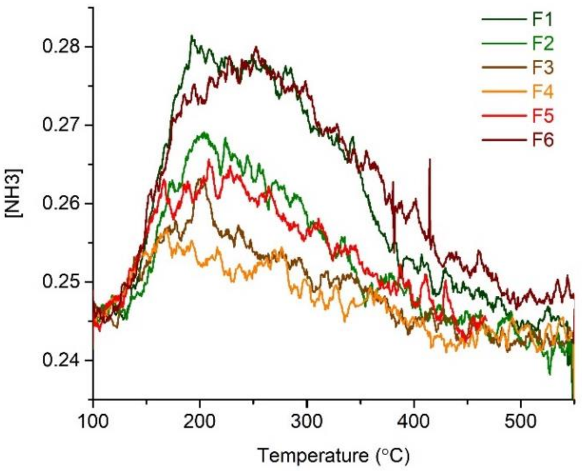

Figure S3: The NH_3-TPD graphs corresponding to the quantified amount of acid sites per density sorted fraction, as shown in Figure 1c.

The FT-IR spectra as depicted in Figure S4, show a decreasing amount of Brønsted acid sites and isolated silanol groups with increasing sample density. Furthermore, it can be observed that strong Lewis acid sites have a low abundance in fractions 1 and 2, but are negligible in higher density fractions. The previously observed decrease in accessibility is also expressed by the amount of physisorbed CO confined in pores: the intensity of this band strongly decreases in fraction 2 in comparison with fraction 1.

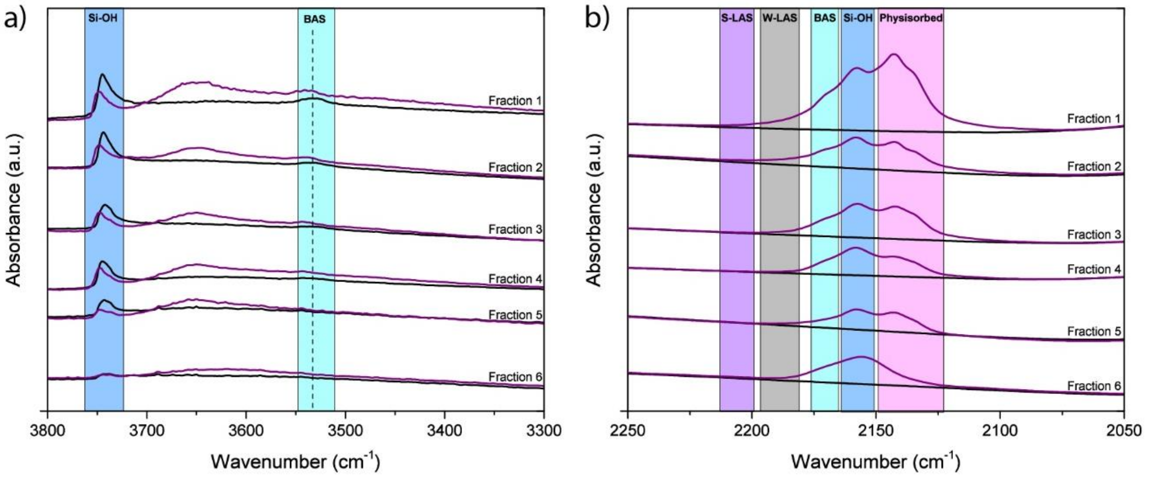

Figure S4: FT-IR spectra collected before (black) and after CO adsorption (purple) for all fractions in the OH vibrational region (a) and CO vibrational region (b) Spectra are corrected for the weight of the self-supporting wafer and plotted with an offset for clarity.

Section S3: single particle screening

Table S3: The amount of particles that was analyzed per density sorted fraction. Depending on the amount of particles in field of view of the X-ray map or fluorescence microscope.

Fraction	# of particles Acidity	# of particles Metals	# of particles Accessibility
**1**	74	229	29
**2**	168	347	76
**3**	218	267	54
**4**	135	463	49
**5**	71	378	54
**6**	101	322	36

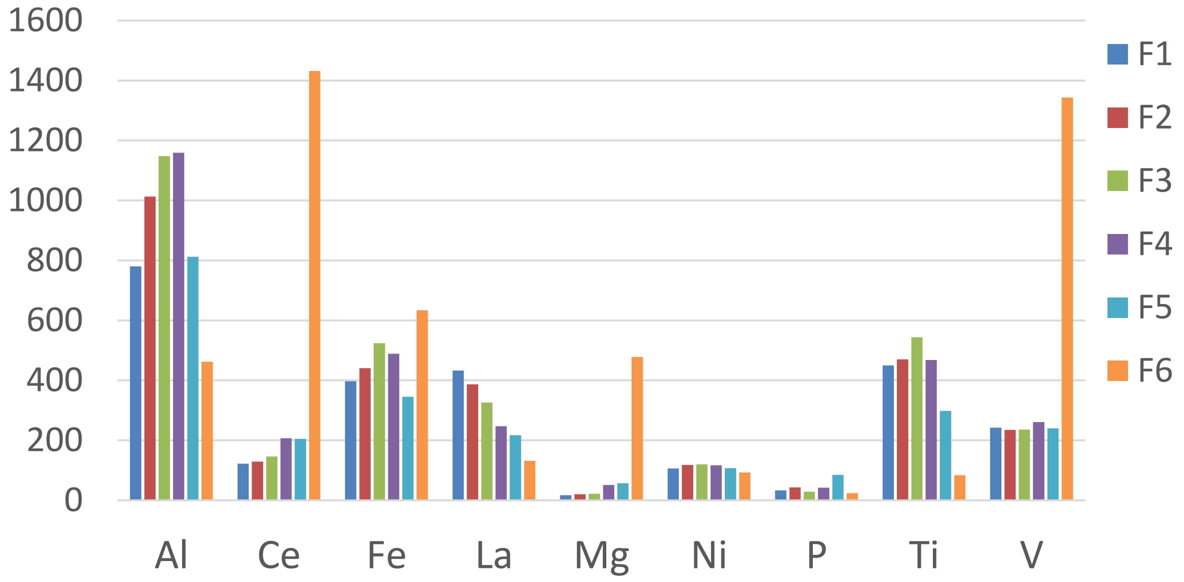

Figure S5: Average XRF values of all elements measured of the sample used for correlations in the main text.

For the XRF experiments, the average values are determined from the complete XRF maps. This includes the background values for every element. All elements that are not shown here, could not be measured with XRF due to the experimental setup.

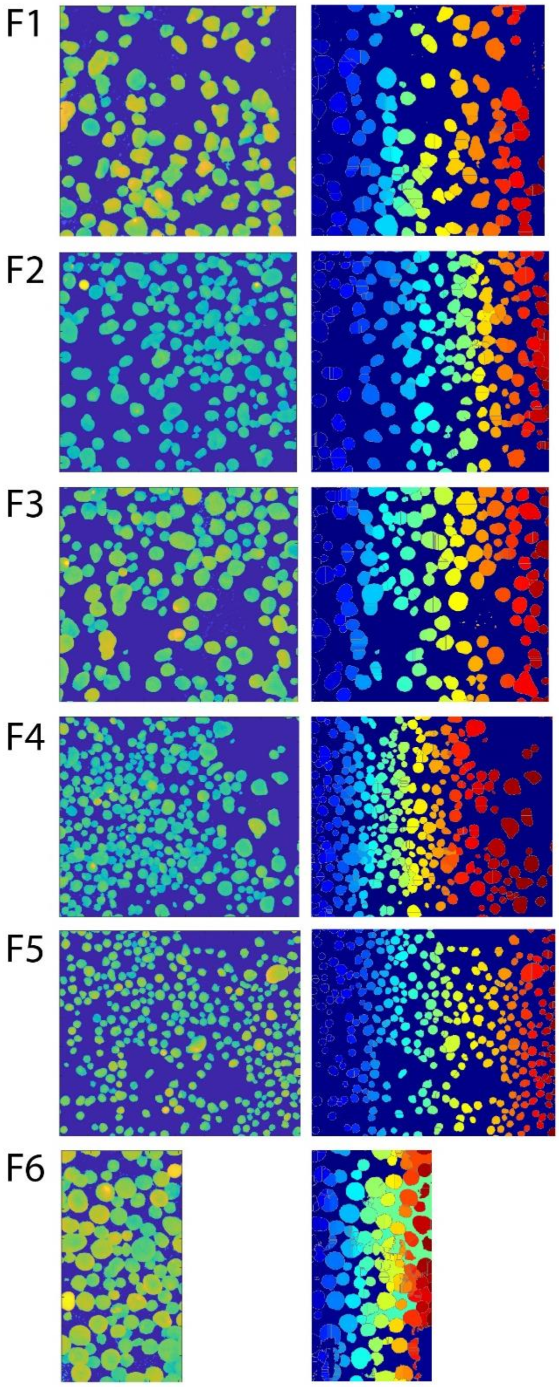

Figure S6: Optical images (left) and corresponding watershed (right) of every density separated fraction.

Single particle segmentation was achieved by a watershed segmentation of the micro-XRF maps. First, corresponding optical images were converted into binary images using adaptive thresholding applying Bradley's method, i.e. by calculating a threshold for each pixel using the local mean intensity around the neighborhood of the pixel.[13] Then, the Euclidean distance transform of the binary image was computed, inverted and the watershed transform was applied. The watershed transform treats an image as a surface where dark pixels represent low and light pixels represent high elevations. In that way the watershed transforms finds so called 'watershed ridge lines' that indicate a separation of regions ('drainage basins') by elevations.[14] When applied to the inverted distance map, the watershed can in this way efficiently separate two overlapping circular objects. Sometimes the watershed shows several separate particles instead of one full large particle, but mostly, the watershed is a good representation of the particles as observed in the micrographs. After the watershed transformation, the identified regions were labeled individually, as displayed in the right part of Figure S7 where each region is indicated by a different color (from dark blue to dark red from left to right in the map). Next, the intensities in each region were determined from the micro-XRF and fluorescence images using the segmented image as a mask. This provided an average XRF/fluorescence intensity for each individual particle identified in the images and enabled the correlation analysis reported in Figures 3 to 7 in the main text. The described approach was implemented using an in-house developed MATLAB code. For fraction F6, one of the segmentations includes a part of the background. This area was excluded from further correlation analyses.

Figure S7 shows that the fluorescence of 4-methoxystyrene is not directly linked to the La content of a particle.

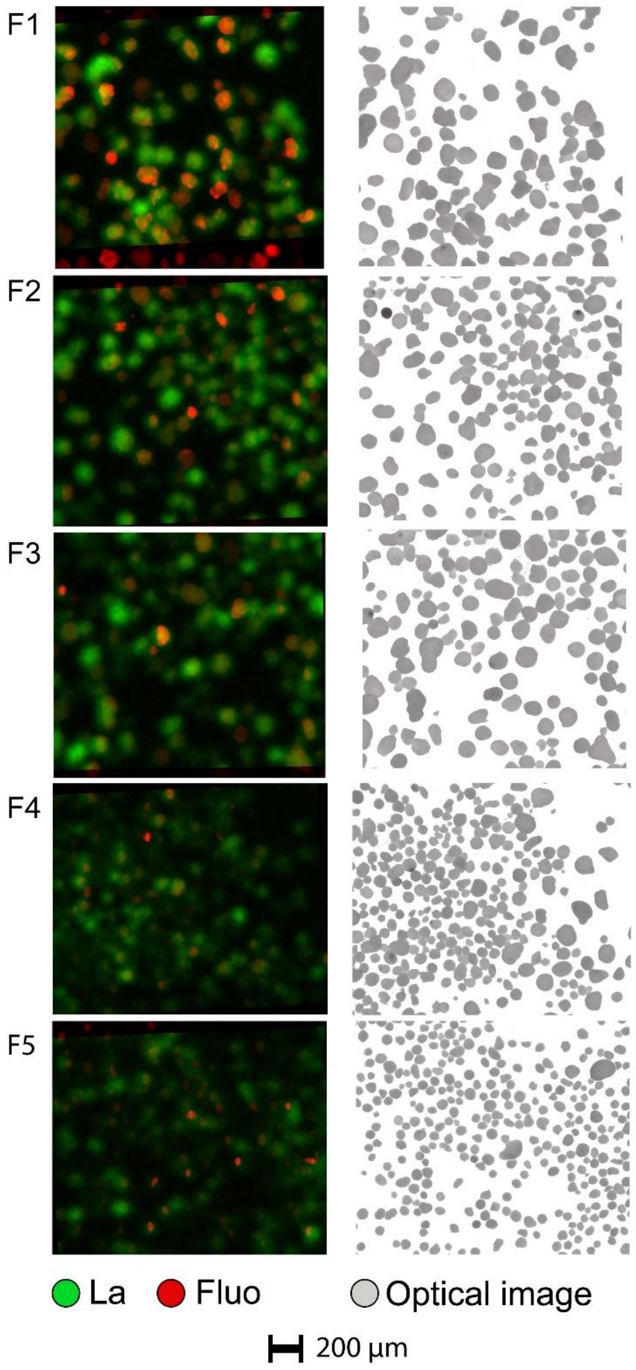

Figure S7: Overlays of styrene fluorescence maps with the La XRF map (F4: little fluorescence that is left comes from La containing particles!) Fraction 6 is left out: due to low fluorescence, this image could not be registered.

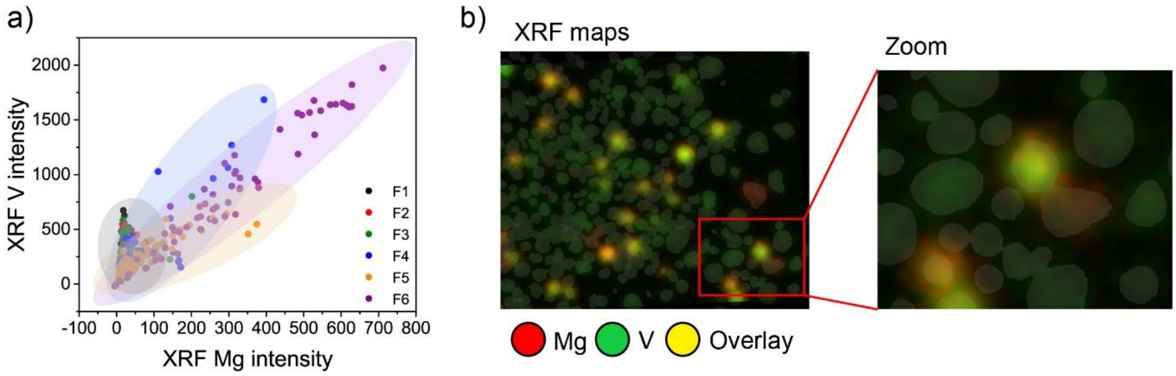

Figure S8: Overlay of V and Mg shows a high correlation, indicating the presence of a Mg based trap for V.

The correlation plot of the XRF maps of Mg and V shows the linear correlation between Mg and V levels. The Pearson correlation coefficients are 0.08, 0.22, 0.32, 0.88 and 0.98 for fractions F1 to F6 respectively. The overlay images of the V and Mg XRF map of fraction 4 together with an optical image (15% opacity) show an example and a zoom onto some Mg based V traps still present in this fraction.

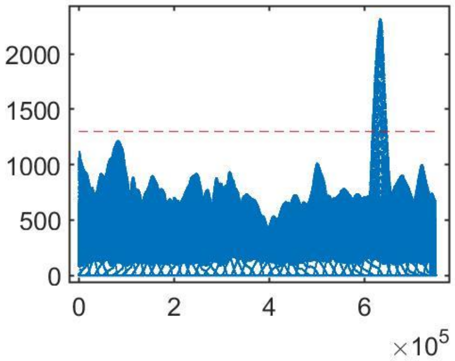

Figure S9: Intensity plot for all pixels (x-axis) in the XRF Fe map of fraction F2 as displayed in Figure 6. The red line shows the cut off value of 1300 used to scale the image. The high Fe particle is an outlier and therefore saturated in the image in Figure 6.

References

[1] M. A. Karreman, I. L. C. Buurmans, A. V Agronskaia, J. W. Geus, H. C. Gerritsen, B. M. Weckhuysen, *Chem. Eur. J.* **2013**, *19*, 3846–3859.
[2] K. Stanciakova, B. Ensing, F. Göltl, R. E. Bulo, B. M. Weckhuysen, *ACS Catal.* **2019**, *9*, 5119–5135.
[3] W. Lutz, *Adv. Mater. Sci. Eng.* **2014**, 724248.
[4] G. R. Dyrkacz, L. Ruscic, C. L. Marshall, W. Reagan, *Energy & Fuels* **2000**, *71*, 849–854.
[5] L. R. Aramburo, S. Wirick, P. S. Miedema, I. L. C. Buurmans, F. M. F. De Groot, B. M. Weckhuysen, *Phys. Chem. Chem. Phys.* **2012**, *14*, 6967–6973.
[6] Z. Ristanović, M. M. Kerssens, A. V. Kubarev, F. C. Hendriks, P. Dedecker, J. Hofkens, M. B. J. Roeffaers, B. M. Weckhuysen, *Angew. Chem. Int. Ed.* **2015**, *54*, 1836–1840.
[7] M. M. Kerssens, A. Wilbers, J. Kramer, P. De Peinder, G. Mesu, *Faraday Discuss.* **2016**, *188*, 69–79.
[8] M. H. F. Kox, E. Stavitski, B. M. Weckhuysen, *Angew. Chem. Int. Ed.* **2007**, *46*, 3652–3655.
[9] J. P. Hofmann, B. M. Weckhuysen, L. R. Aramburo, J. Ruiz-Martínez, *Catal. Sci. Technol.* **2013**, *3*, 1208–1214.
[10] Z. Ristanović, J. P. Hofmann, G. De Cremer, A. V. Kubarev, M. Rohnke, F. Meirer, J. Hofkens, M. B. J. Roeffaers, B. M. Weckhuysen, *J. Am. Chem. Soc.* **2015**, *137*, 6559–6568.
[11] I. L. C. Buurmans, J. Ruiz-Martínez, W. V Knowles, D. van der Beek, J. A. Bergwerff, E. T. C. Vogt, B. M. Weckhuysen, *Nat. Chem.* **2011**, *3*, 862–867.
[12] K. Janssens, in *Handb. Spectrosc.* (Eds.: G. Gauglitz, D.S. Moore), Wiley-VCH Weinheim, **2014**, pp. 451–508.
[13] D. Bradley, G. Roth, *J. Graph. Tools* **2007**, *12*, 13–21.
[14] F. Meyer, *Signal Processing* **1994**, *38*, 113–125.
